# Biomechanical Study of the Preventive Effect of Different Cephalomedullary Fixation Methods on the Occurrence of Femoral Neck Fractures in Osteoporotic Femurs

**DOI:** 10.3390/medicina62081560

**Published:** 2026-08-14

**Authors:** Incheol Kook, Ki-Chul Park, Hyoung Keun Oh, Je-Hyun Yoo, Chang-Nam Kang, Kyu Tae Hwang

**Affiliations:** 1 Department of Orthopedic Surgery, Cheongju TOP Hospital, 1 Gyoseo-ro, Cheongju 28546, Republic of Korea; nathan0319@naver.com; 2 Department of Orthopedic Surgery, Hanyang University Guri Hospital, Guri 11923, Republic of Korea; kcpark@hanyang.ac.kr; 3 Department of Orthopedic Surgery, Ilsan Paik Hospital, Inje University College of Medicine, Goyang 10380, Republic of Korea; ilsanosd11@gmail.com; 4 Department of Orthopaedic Surgery, Hallym University Sacred Heart Hospital, Hallym University College of Medicine, Anyang 14068, Republic of Korea; oships@hallym.ac.kr; 5 Department of Orthopaedic Surgery, Hanyang University Seoul Hospital, 222 Wangsimni-ro, Seoul 07345, Republic of Korea; cnkang65@hanyang.ac.kr

**Keywords:** cephalomedullary fixation, osteoporotic femur, biomechanical study, peri-implant fracture, intramedullary nailing

## Abstract

*Background and Objectives:* This study aimed to investigate the biomechanical properties of different cephalomedullary fixation methods for osteoporotic femurs using a synthetic bone model. *Materials and Methods:* Lateral impact tests and axial cyclic loading tests were performed, and each test was divided into three groups: group 1 with one reconstruction screw, group 2 with two reconstruction screws, and group 3 without cephalomedullary fixation. To simulate a healed femoral shaft fracture, no osteotomy was performed on the specimens. *Results:* Groups 1 and 2 had significantly higher mean stiffness, ultimate failure load, and energy to failure in the lateral impact test compared to the native synthetic femur (*p* < 0.001 for all), while group 3 had significantly lower values in all outcomes (*p* < 0.05 for all). Groups 1 and 2 showed significantly higher average number of cycles, total load to failure, and initial axial stiffness in the axial cyclic incremental loading test compared to the native synthetic femur (*p* < 0.001 for all), whereas group 3 showed a significantly lower mean number of cycles and total load to failure (*p* < 0.001 for all). *Conclusions:* Under the specific test conditions of this intact synthetic bone model, cephalomedullary fixation with one or two reconstruction screws demonstrated superior relative mechanical stability compared to standard interlocking nails. Using two reconstruction screws provided the greatest biomechanical resistance to failure. In cases where inserting two reconstruction screws is technically challenging or infeasible, a single reconstruction screw may offer an acceptable biomechanical alternative to help prevent peri-implant fractures in healed or prophylactic settings. Intramedullary nailing without cephalomedullary fixation exhibited the lowest mechanical performance, suggesting a potential biomechanical vulnerability to peri-implant fractures under these test conditions.

## 1. Introduction

Femoral shaft fractures in elderly patients are predominantly caused by low-energy injuries [1,2]. In elderly patients, the progression of osteoporosis and femoral bowing increases the risk of femoral shaft fractures, including subtrochanteric fractures, and the increased risk of falls due to advanced age and frailty also contributes to the development of these fractures [3,4,5,6,7,8,9].

The standard treatment for femoral shaft fractures is intramedullary (IM) nailing, and methods for proximal fixation of the IM nail include inserting a standard interlocking screw into the trochanteric area or performing cephalomedullary fixation by inserting a reconstruction screw that extends through the femoral neck to the head [10,11]. While the use of standard interlocking screws offers the advantages of a simplified technique and shorter operative time, a major drawback is that the screw inserted into the trochanteric region can act as a stress riser [12,13,14]. Consequently, in the event of a secondary fall, standard interlocking fixation fails to provide additional protection to the femoral neck and may actually predispose the osteoporotic bone to proximal peri-implant fractures [12,13,14].

Alternatively, cephalomedullary fixation typically involves the parallel insertion of two reconstruction screws. Although this configuration provides robust secondary protection to the femoral neck, it has notable limitations. The procedure can prolong surgical time and increase radiation exposure due to the precision required to establish safe trajectories within the femoral neck [15,16]. Furthermore, inserting two screws can be technically challenging or even unfeasible in patients with an anatomically narrow femoral neck [17,18,19,20,21].

As a practical compromise, a single reconstruction screw can be inserted and is currently used in clinical practice [22,23]. Although cephalomedullary fixation with a single reconstruction screw is believed to provide sufficient femoral neck protection and prevent proximal peri-implant fractures, its biomechanical properties have not been elucidated. To the best of our knowledge, there have been no studies to date reporting on the biomechanical differences between using one reconstruction screw and two reconstruction screws for cephalomedullary fixation of a femoral IM nail. Identifying the biomechanical characteristics and extent of femoral neck protection provided by each cephalomedullary fixation method will be helpful in determining treatment options for osteoporotic femoral shaft fractures.

This study aimed to investigate the biomechanical characteristics of different cephalomedullary fixation methods (using one or two reconstruction screws) of IM nails using an osteoporotic synthetic femur. We also sought to determine the biomechanical differences between cephalomedullary and non-cephalomedullary fixations.

## 2. Materials and Methods

### 2.1. Specimen Preparation

A synthetic femur bone model (Sawbones, Pacific Research Laboratories, Vashon, WA, USA) covered with a thin cortical layer and a cancellous region filled with polyurethane foam at a density corresponding to that of human osteoporotic bone was used in this biomechanical study. The femoral model was 470 mm long, with a neck-shaft angle of 125°, anteversion of 15°, head diameter of 48 mm, and canal diameter of 16 mm. The canal diameter of the model was set to be large to reflect medullary expansion in elderly femur [24,25].

The study group was divided into three groups: IM nailing with cephalomedullary fixation using one reconstruction screw (group 1), IM nailing with cephalomedullary fixation using two reconstruction screws (group 2), and IM nailing without cephalomedullary fixation using proximal standard interlocking screws (group 3). A power analysis based on a previous biomechanical study of the effectiveness of hip fracture prevention following implantation of a cephalomedullary device in elderly cadaveric femurs showed that a sample size of seven per group was sufficient to achieve 80% power at a significance level of 0.1 [26].

The IM nail implant used in this study was a Femoral Recon Nail System (DePuy Synthes, Raynham, MA, USA). The nail system was a greater trochanteric (GT) entry nail with a length of 360 mm, diameter of 10 mm, and a neck-shaft angle of 130°.

A guide pin was placed at the GT tip of the femoral model and inserted along the C-arm guide to center on the proximal shaft in anteroposterior (AP) and lateral views. An entry portal was created along the inserted guide pin, after which the IM nail was attached to a targeting device and inserted into the synthetic femur. Because the canal diameter was sufficiently larger than the diameter of the IM nail, medullary reaming was unnecessary.

**Figure 1:**
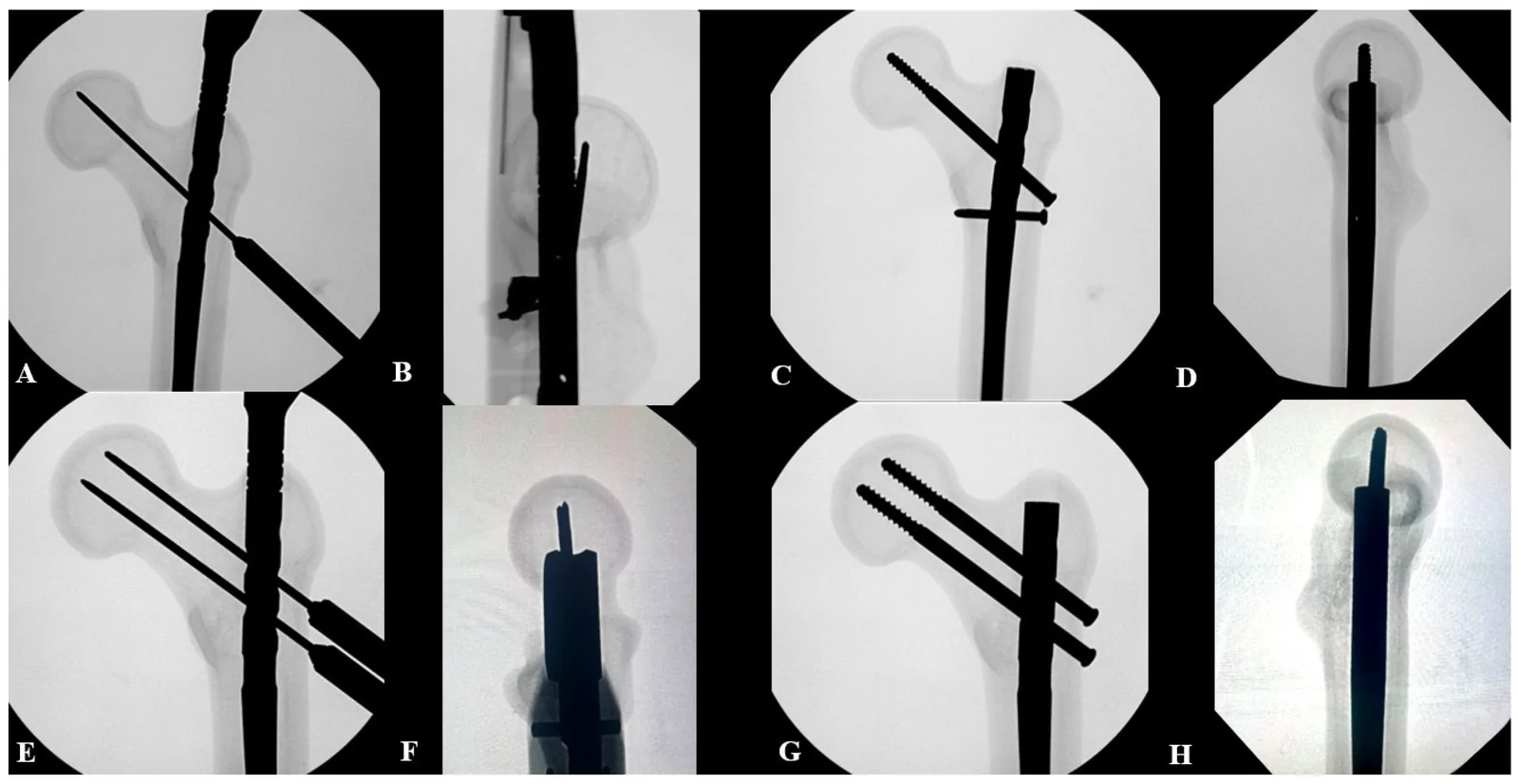
Anteroposterior (**A**) and lateral (**B**) C-arm images of cephalomedullary fixation with one reconstruction screw. A guide pin was inserted through the inferior cephalomedullary slot using a jig connected to an intramedullary (IM) nail. Anteroposterior (**C**) and lateral (**D**) C-arm images obtained after IM nailing. The reconstruction screw is positioned in a center-to-center position, with the proximal end of the IM nail slightly below the greater trochanter tip. Anteroposterior (**E**) and lateral (**F**) C-arm images of cephalomedullary fixation with two reconstruction screws. Guide pins were inserted in parallel through the superior and inferior cephalomedullary slots, using a targeting device connected to an IM nail. Anteroposterior (**G**) and lateral (**H**) views after IM nailing with two reconstruction screws. Note that the midpoints of the two reconstruction screws are in a center-to-center position, and the IM nail was inserted deeply below the greater trochanter tip.

Cephalomedullary fixation using a single reconstruction screw was initiated by inserting a guide pin into the femoral head through the lower cephalomedullary slot of the jig. The inserted guide pin was then confirmed through the C-arm to be located at the center of the femoral head in both the AP and lateral views. The guide pin tip was 5–10 mm below the cortex of the femoral head apex, and the reconstruction screw was inserted after drilling to an appropriate length. The reconstruction screw was inserted through the lower slot to prevent placement of the IM nail too deeply and hollowing out the GT tip. Consequently, the IM nail was placed approximately 5 mm below the GT tip. After inserting the reconstruction screw, one transverse interlocking screw was inserted into the lesser trochanter area (Figure 1).

Cephalomedullary fixation with two reconstruction screws was performed by inserting two parallel guide pins into the femoral head. The midpoint of the two guide pins was ensured to be located at the center of the femoral head on the AP and lateral views through the C-arm. The guide pin tip was placed 5–10 mm inferior to the femoral head cortex along the trajectory of each guide pin, and a reconstruction screw of appropriate length was inserted. By placing the two reconstruction screws, with their midpoint center-to-center on the femoral head, the discrepancy between the neck-shaft angle of the synthetic femur bone and the IM nail allowed the IM nail to be inserted deeper; thus, the proximal end of the nail was 10 mm below the GT tip (Figure 1).

**Figure 2:**
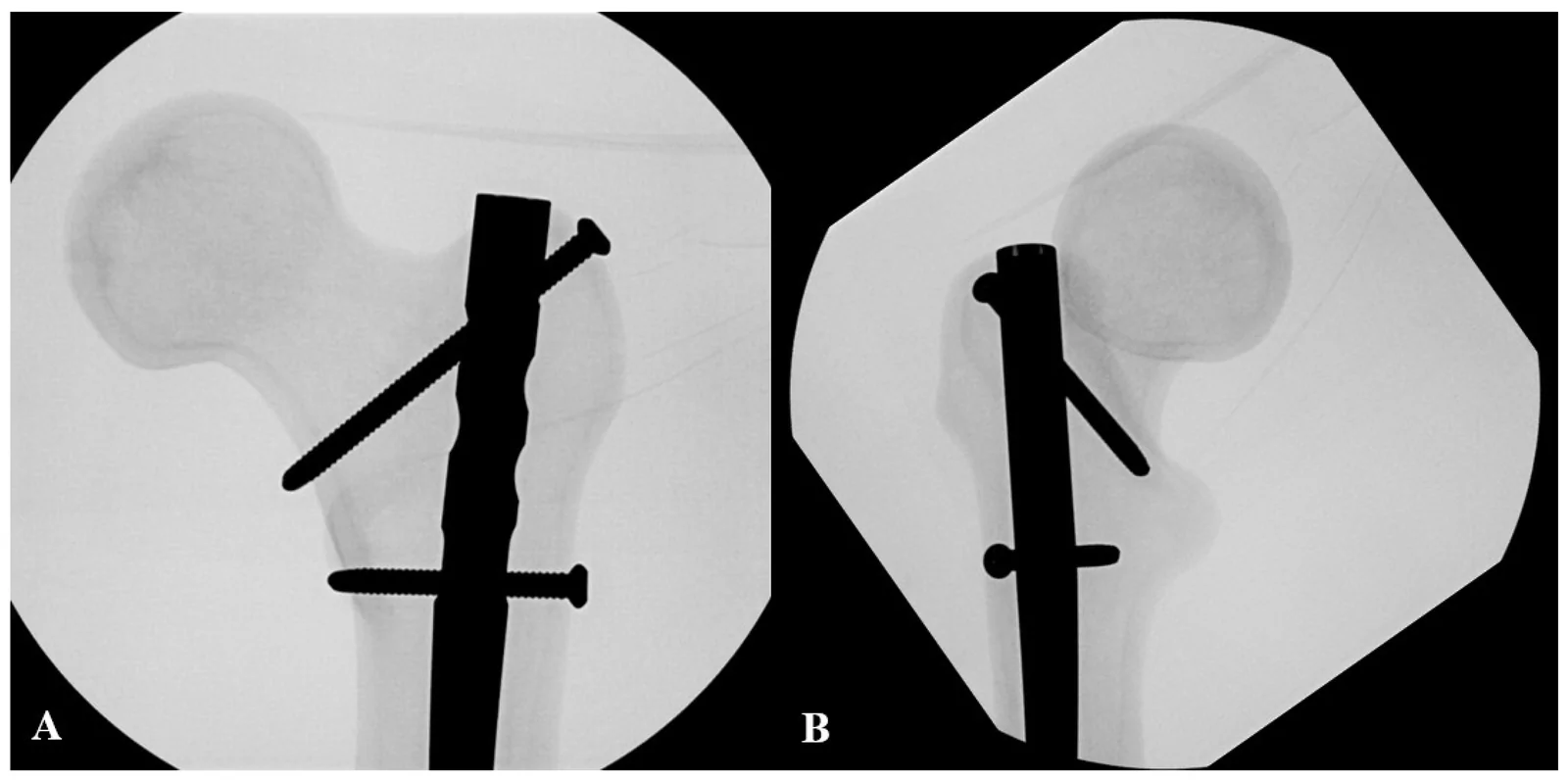
Oblique and transverse interlocking screws were inserted using a targeting device connected to an intramedullary (IM) nail. Anteroposterior (**A**) and lateral (**B**) C-arm images after IM nailing with standard interlocking screws.

Standard proximal interlocking screws were inserted using a customized jig. First, an oblique screw was inserted after drilling across the trochanteric area using a jig. Next, a transverse screw was placed under the oblique screw. The proximal end of the IM nail was flush with the GT tip (Figure 2). All IM nails were statically locked by inserting two distal interlocking screws. A proximal end cap was not used in any case.

### 2.2. Biomechanical Test Protocol

Two types of biomechanical tests were performed: a lateral impact test and an axial cyclic incremental loading test. Seven samples per group were assigned for each test. Biomechanical tests were performed on an electrodynamic testing machine (Instron e10000; Instron, Norwood, MA, USA) with a load cell capacity of 10 kN. Load (N) and displacement (mm) data were collected at a rate of 100 Hz using a transducer attached to the testing machine, and a load–displacement curve was plotted based on the data.

**Figure 3:**
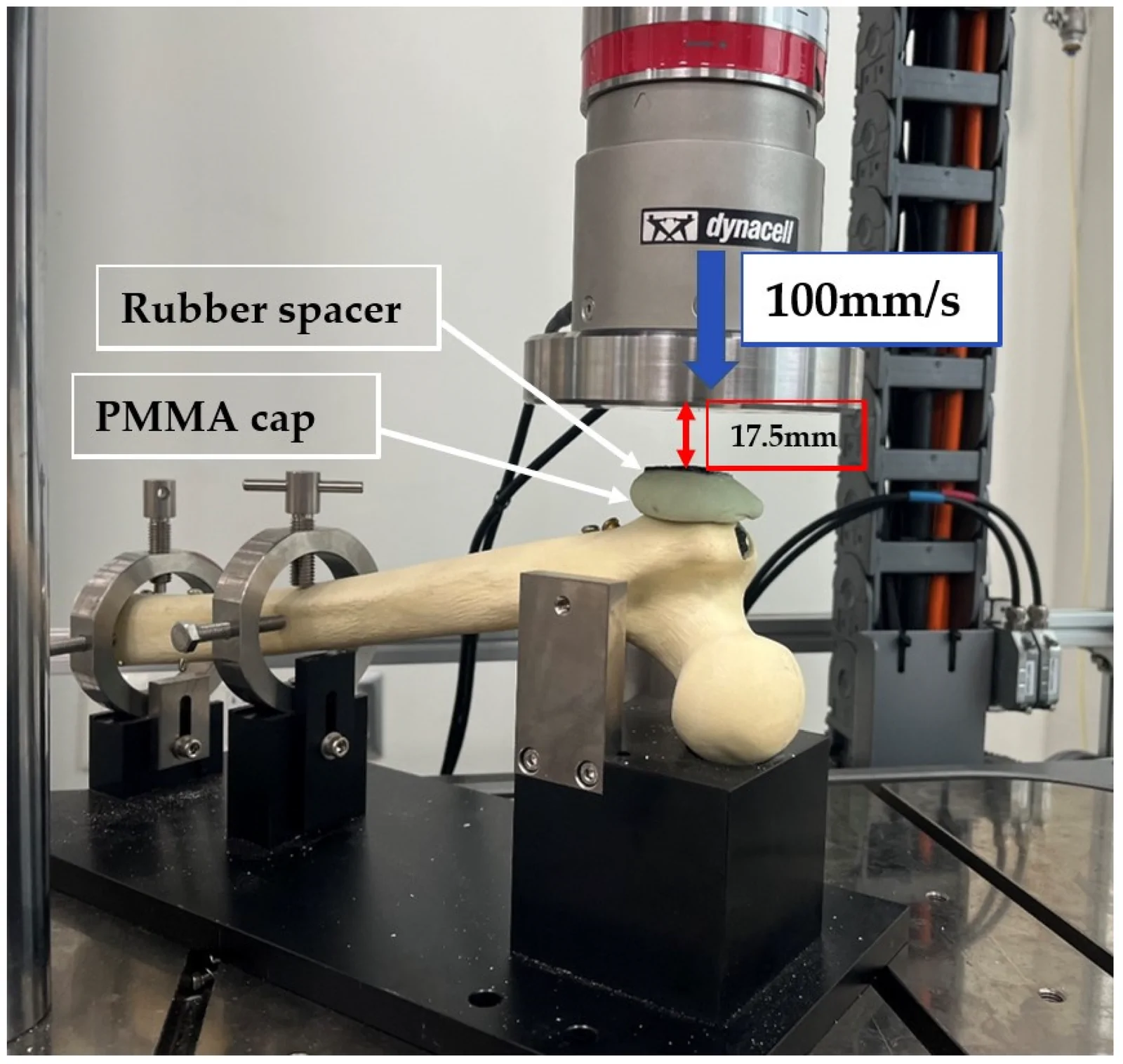
Setup for the lateral impact test. The blue arrow indicates the direction and velocity (100 mm/s) of the applied load, and the red double-headed arrow indicates the initial distance (17.5 mm) between the load cell and the specimen.

The lateral impact test was conducted because more than 90% of hip fractures in the elderly are related to falls, and studies have shown that falling sideways and landing on the lateral side of the hip increases the risk of hip fracture by approximately 20 times compared to falling in the other direction [27,28,29,30]. The lateral impact test followed the protocol of previous studies and involved applying a load to the lateral aspect of the greater trochanter at a constant speed of 100 mm/s, with the axis of the femoral shaft at 10° to the horizontal plane and anteversion of the femoral neck at 15° [26,31,32]. The distal 8 cm of the specimen was removed using an oscillating saw to clamp the specimen in the jig, with the femoral shaft inclined at 10° from the horizontal plane. Since the angle between the anatomical axis of the femoral shaft and the horizontal line is 10°, inclining the femoral shaft by 10° compensates for this alignment, allowing the impact force to be applied to the femur from a true-lateral direction. After clamping the specimen, a cap made of polymethylmethacrylate (PMMA; Palacos R, Heraeus, Hanau, Germany) was placed on the lateral side of the GT to distribute the load. A thin rubber spacer was attached over the cap. A vertical jig attached to the load cell was positioned 17.5 mm above the rubber spacer. The vertical jig was then descended at a constant speed of 100 mm/s, and the specimen was loaded until failure occurred (Figure 3). The outcomes of the lateral impact test were stiffness, ultimate failure load, and energy to failure. The slope of the elastic stage of the load–displacement curve was calculated to define stiffness (N/mm). Failure was defined as cut-out of the implant, implant failure, fracture of the femoral neck or shaft, or a sudden drop in load of >30% [33]. The force immediately before the point where the load suddenly dropped by >30% and did not recover in was defined as the ultimate failure load (N), and the area under the load–displacement curve up to the ultimate failure load was defined as the failure energy (J) [33,34,35]. After the lateral impact test, C-arm was used to determine the failure patterns and fracture configurations in each group.

**Figure 4:**
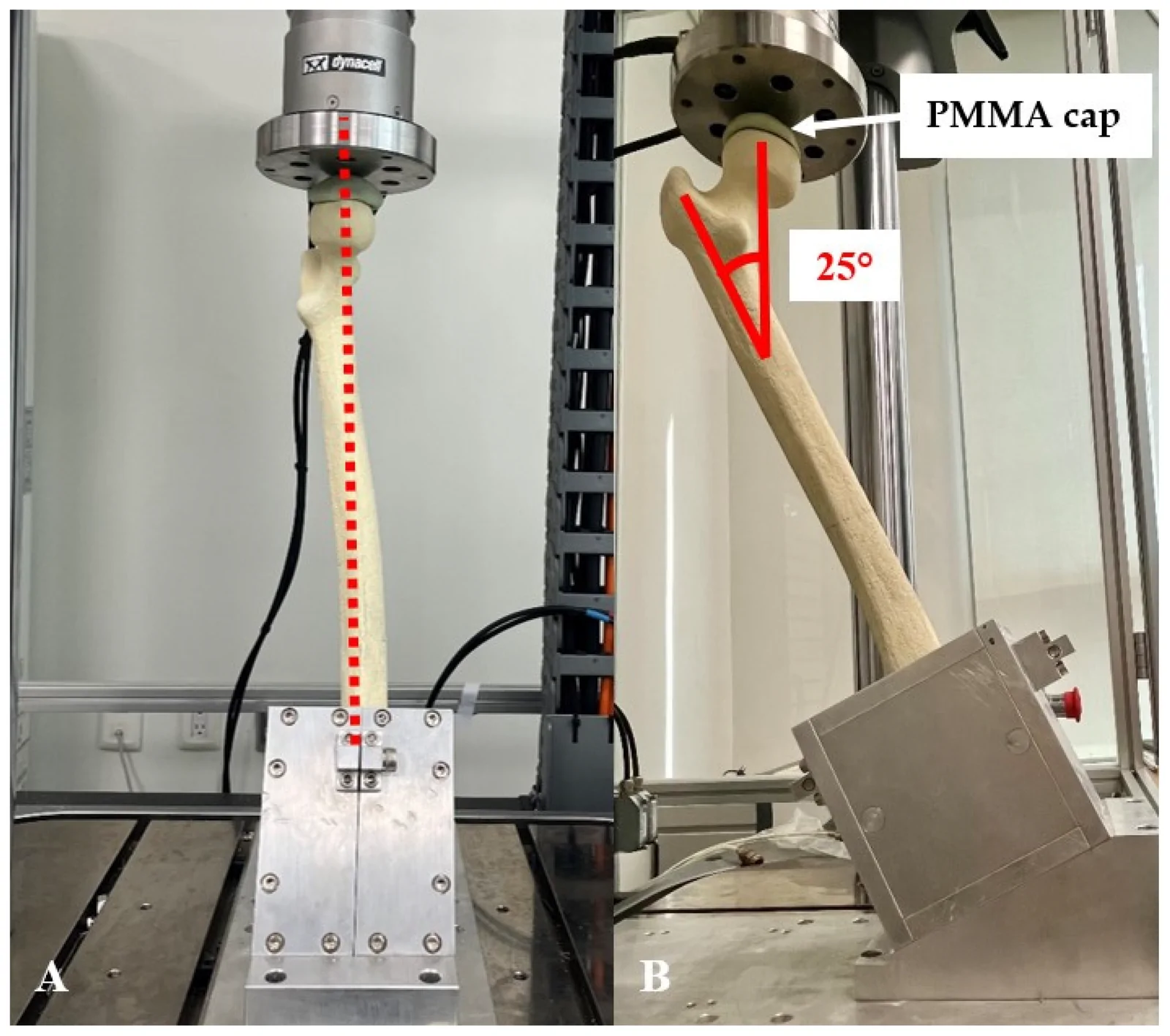
Anteroposterior (**A**) and lateral (**B**) setup for the axial cyclic incremental loading test. The specimen is positioned neutral in the sagittal plane and 25° adducted in the coronal plane to simulate a single-leg stance. The red dashed line in (**A**) represents the vertical loading axis, and the red marks in (**B**) illustrate the 25° adduction angle. The label also indicates the position of the PMMA cap.

An axial cyclic incremental loading test was performed in accordance with the protocol in previous studies [33,36]. The specimen was positioned in a customized metal square jig with a neutral position in the coronal and sagittal planes and fixed by pouring resin at the distal 8 cm [33]. The area fixed with resin was inferior to the distal tip of the IM nail. The square metal jig holding the specimen was connected to the lower base jig, which was designed to have a 25° slope in the coronal plane. Eventually, the specimen was positioned at 25° adduction in the coronal plane. The position of the specimen simulated an anatomical single-leg stance [37,38,39] (Figure 4). The femoral head was covered with a PMMA cap to simulate the acetabulum. The specimen was mounted on an electrodynamic testing machine, and a preload of 35 N was applied at a rate of 3.5 N/s, followed by a cyclic axial load [39] (Figure 4). The maximum axial load was increased from 350 N to 2800 N in increments of 175 N every 10,000 cycles, assuming a 70 kg (approximately 700 N) patient initiating partial weight bearing at a level of 50% of body weight and progressively increasing the load and eventually standing on one leg unassisted with a force of approximately 400% of body weight applied to the hip joint [36,40,41]. Cyclic loading was performed in a sinusoidal pattern from 10% to 100% of the corresponding maximum axial load at a frequency of 3 Hz [36]. Failure in the axial cyclic incremental loading test was defined as cut-out of the implant, implant failure, fracture of the femoral neck or shaft, displacement greater than 15 mm, or a sudden drop in load > 30% [33,34]. The outcomes of the axial loading test included the number of cycles, total load applied to failure (kN), and initial axial stiffness (N/mm). Initial axial stiffness was determined after three loading cycles to account for the settling effect of the specimen [39]. C-arm image was obtained after the axial cyclic incremental loading test to determine the failure mode and fracture morphology for each group.

Since the purpose of this study was to investigate the occurrence of secondary fractures and the stability of the implant structure during a secondary sideways fall or progressive weight-bearing following the treatment of a femoral shaft fracture, it was necessary to simulate a condition in which the fracture had completely healed, rather than the acute “time zero” state immediately post-fixation. Furthermore, creating a femoral shaft osteotomy introduces significant confounding variables—such as variations in the fracture gap and cortical contact—which could complicate the interpretation of the biomechanical results at the proximal femur. Therefore, intact synthetic femurs were utilized in this study, under the assumption that the diaphyseal fracture had already united.

Preliminary experiments were conducted on the lateral impact and axial cyclic incremental loading tests to determine the biomechanical properties of the native synthetic femur used in this study. Seven specimens were used for each test, and the preliminary results showed that the average stiffness, ultimate failure load, and energy to failure of the native synthetic femur in the lateral impact test were 274.5 ± 4.3 N/mm, 1932.3 ± 39.4 N, and 9.0 ± 0.4 J, respectively. The average number of cycles, total load, and initial axial stiffness of the native synthetic femur in the axial cyclic incremental loading test were 54,894.6 ± 1110.6 cycles, 40,995.9 ± 1360.5 kN, and 282.5 ± 5.9 N/mm, respectively.

### 2.3. Statistical Analysis

A comparative analysis was performed between the three groups. Continuous variables were tested for normality using the Shapiro–Wilk test. Once normality was satisfied, either analysis of variance (ANOVA) and Tukey’s honestly significant difference (HSD) post hoc analysis or Welch’s ANOVA and Games–Howell post hoc analysis were performed according to the results of Levene’s test of homogeneity. For nonparametric data, Kruskal–Wallis test and Dunn’s post hoc analysis were used. The biomechanical properties of each group were compared with those of the native synthetic femur. Independent *t*-tests were used for variables that met normality; otherwise Mann–Whitney U tests were performed. All statistical analyses were performed using SPSS software (version 27.0; IBM Corp., Armonk, NY, USA). Statistical significance was set at *p* < 0.05, and Bonferroni’s correction was used for post hoc analysis to set statistical significance at a *p* < 0.017.

### 2.4. Use of Artificial Intelligence

Generative AI (Google’s Gemini 3.1 Pro) was utilized solely for the purpose of language editing, proofreading, and improving the grammatical flow of the manuscript during its preparation. The AI tool was not used to generate any scientific data, ideas, or conclusions, and the authors remain fully responsible for the entire content and integrity of the submitted paper.

## 3. Results

**Figure 5:**
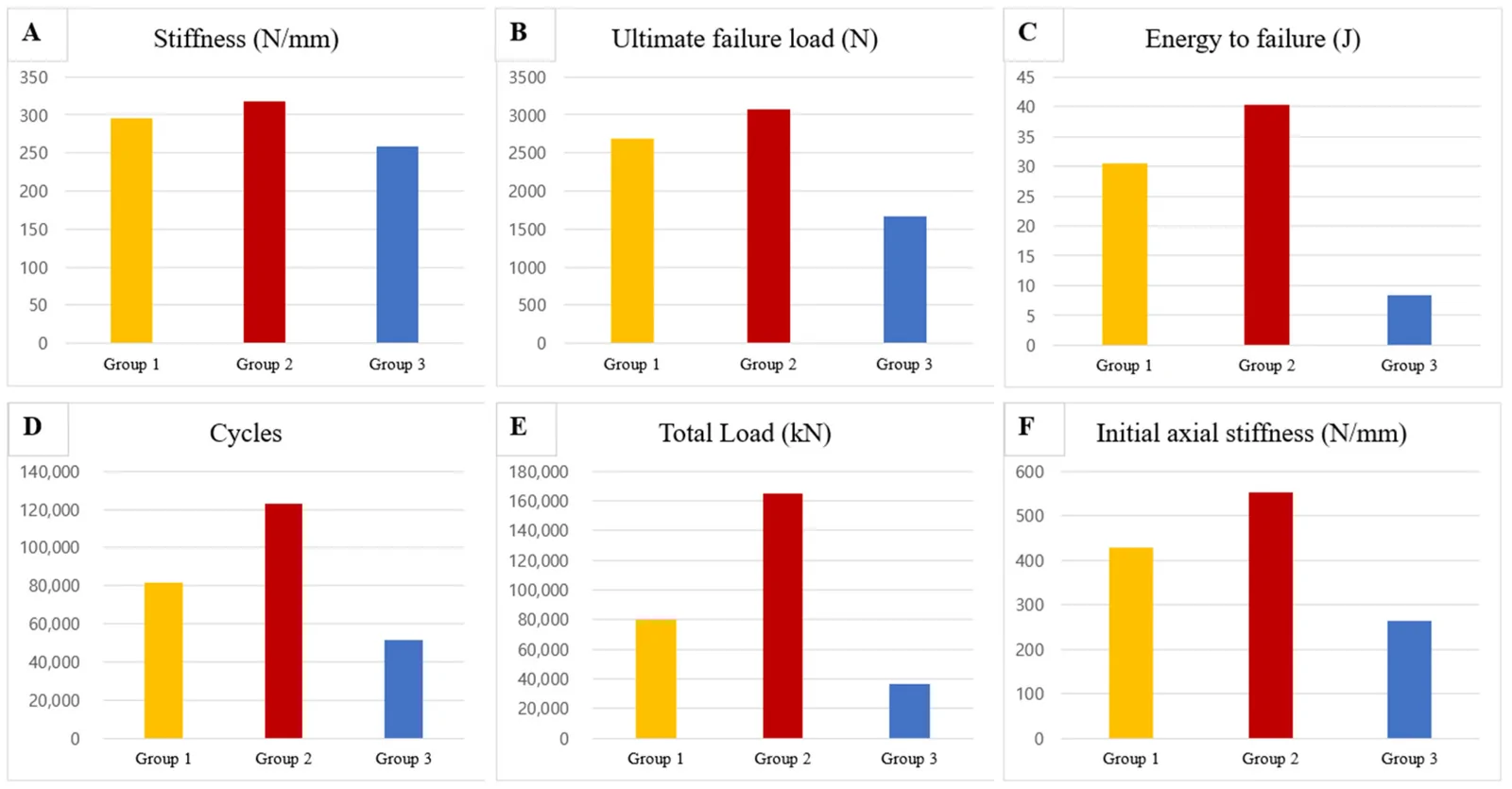
Graphs showing the outcomes of the lateral impact test (**A**–**C**) and axial incremental loading test (**D**–**F**) for each group. (**A**) Stiffness. (**B**) Ultimate failure load. (**C**) Energy to failure. (**D**) Number of cycles. (**E**) Total Load. (**F**) Initial axial stiffness. Significant differences of *p* < 0.001 are observed between the groups for each outcome.

The mean stiffness in the lateral impact test was in the order of group 2 (317.5 ± 6.2 N/mm), group 1 (296.0 ± 5.0 N/mm), and group 3 (258.6 ± 3.8 N/mm), and showed significant differences between the three groups (*p* < 0.001, effect size η^2^ = 0.964, 95% confidence interval [CI]: 0.910–0.976) (Figure 5). Post hoc analysis using Tukey’s HSD test revealed significant differences across all direct pairwise comparisons. Specifically, group 2 demonstrated significantly greater stiffness than group 1 (*p* < 0.001), and group 1 showed significantly greater stiffness than group 3 (*p* < 0.001).

Similarly, the mean ultimate failure load and energy to failure followed the same order: group 2 (3074.6 ± 173.5 N, 40.4 ± 1.4 J), group 1 (2681.4 ± 92.8 N, 30.4 ± 1.4 J), and group 3 (1671.8 ± 29.8 N, 8.4 ± 0.1 J). The omnibus tests indicated significant differences among the groups for both ultimate failure load (*p* < 0.001, effect size η^2^ = 0.969, 95% CI: 0.921–0.979) and energy to failure (*p* < 0.001, effect size η^2^ = 0.993, 95% CI: 0.983–0.996) (Figure 5). Subsequent direct comparisons via Tukey’s HSD test confirmed that group 2 had significantly higher ultimate failure load and energy to failure compared to group 1 (both *p* < 0.001). Additionally, group 1 exhibited significantly higher values in both parameters compared to group 3 (both *p* < 0.001).

Furthermore, independent *t*-tests were conducted to compare the biomechanical properties of each fixation construct against the native synthetic femur. Groups 1 and 2 showed significantly greater stiffness, ultimate failure load, and energy to failure compared to the native synthetic femur (all *p* < 0.001). The mean stiffness (*p* < 0.001), ultimate failure load (*p* < 0.001), and energy to failure (*p* = 0.013) in group 3 were 5.8%, 13.5%, and 6.7% lower, respectively, than those of native synthetic femur, with significant differences(Table 1).

**Table 1:** Biomechanical results of the lateral impact and axial cyclic loading tests for each fixation group and the control group (native synthetic femur).

	Control (Native Synthetic Femur)	Group 1 (1 Reconstruction Screw)	Group 2 (2 Reconstruction Screws)	Group 3 (Standard Interlocking)
Stiffness (N/mm)	274.5 ± 4.3	296.0 ± 5.0 (+7.8%) *	317.5 ± 6.2 (+15.7%) *	258.6 ± 3.8 (−5.8%) *
Ultimate failure load (N)	1932.3 ± 39.4	2681.4 ± 92.8 (+38.8%) *	3074.6 ± 173.5 (+59.1%) *	1671.8 ± 29.8 (−13.5%) *
Energy to failure (J)	9.0 ± 0.4	30.4 ± 1.4 (+237.8%) *	40.4 ± 1.4 (+348.9%) *	8.4 ± 0.1 (−6.7%) *
Cycles	54,894.6 ± 1110.6	81,531.4 ± 616.0 (+48.5%) *	122,918.7 ± 1117.4 (+123.9%) *	51,618.0 ± 569.1 (−6.0%) *
Total Load (kN)	40,995.9 ± 1360.5	79,680.0 ± 1078.0 (+94.3%) *	164,650.9 ± 2737.7 (+301.6%) *	36,982.1 ± 697.2 (−9.8%) *
Initial axial stiffness (N/mm)	282.5 ± 5.9	427.9 ± 8.1 (+51.4%) *	553.6 ± 19.4 (+95.7%) *	264.5 ± 9.7 (−6.4%) *

Data are presented as mean ± standard deviation. * Values indicate the percentage change (increase or decrease) relative to the control group.

**Figure 6:**
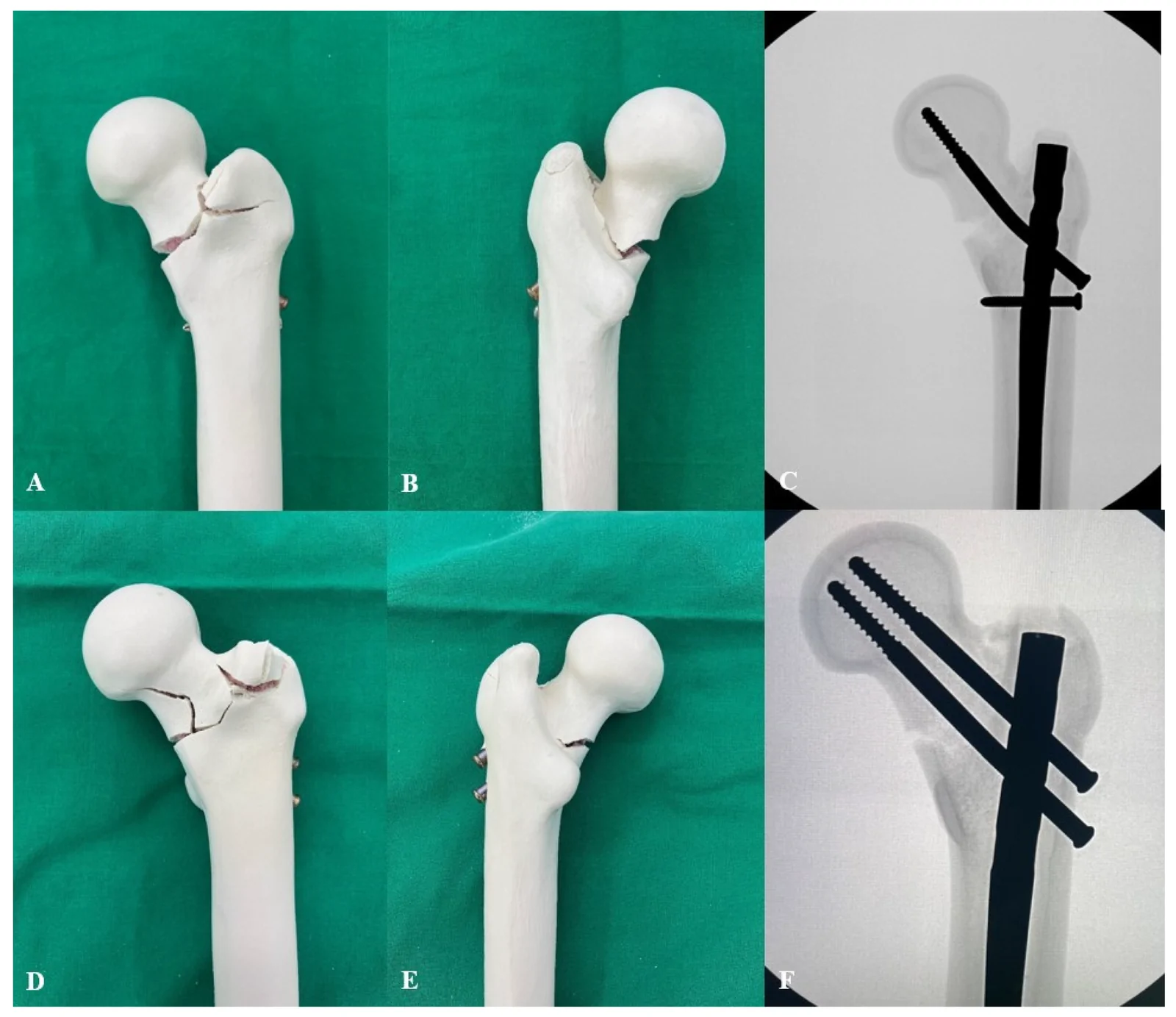
Anterior (**A**) and posterior (**B**) views of the specimen after the lateral impact test in group 1. Distraction of the medial femoral neck, compression of the lateral femoral neck, and upward bending of the reconstruction screws are observed in the anteroposterior C-arm image (**C**). Anterior (**D**) and posterior (**E**) views of the specimen after the lateral impact test in group 2. In the anteroposterior C-arm image (**F**), slight upward bending of the lower reconstruction screw is observed.

**Figure 7:**
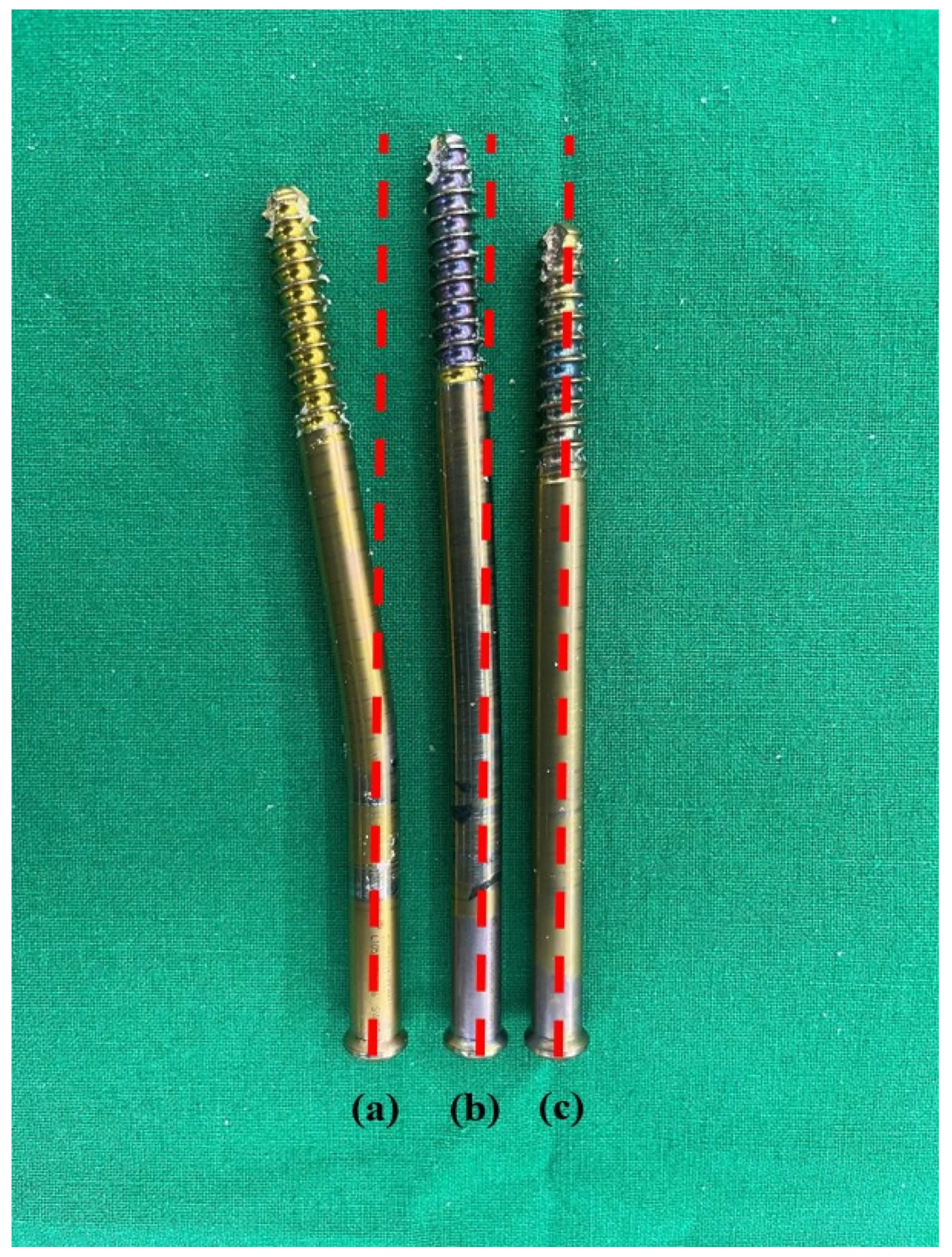
Photographs of the reconstruction screws in groups 1 and 2 after the lateral impact test. Single reconstruction screw (**a**) in group 1, lower reconstruction screw (**b**) in group 2, and upper reconstruction screw (**c**) in group 2. The red dashed lines represent the straight longitudinal axis drawn through the distal part of each screw body, serving as a reference to visually highlight the degree of bending. Note that the screws are bent in the following order: single reconstruction screw in group 1, lower reconstruction screw in group 2, and upper reconstruction screw in group 2.

**Figure 8:**
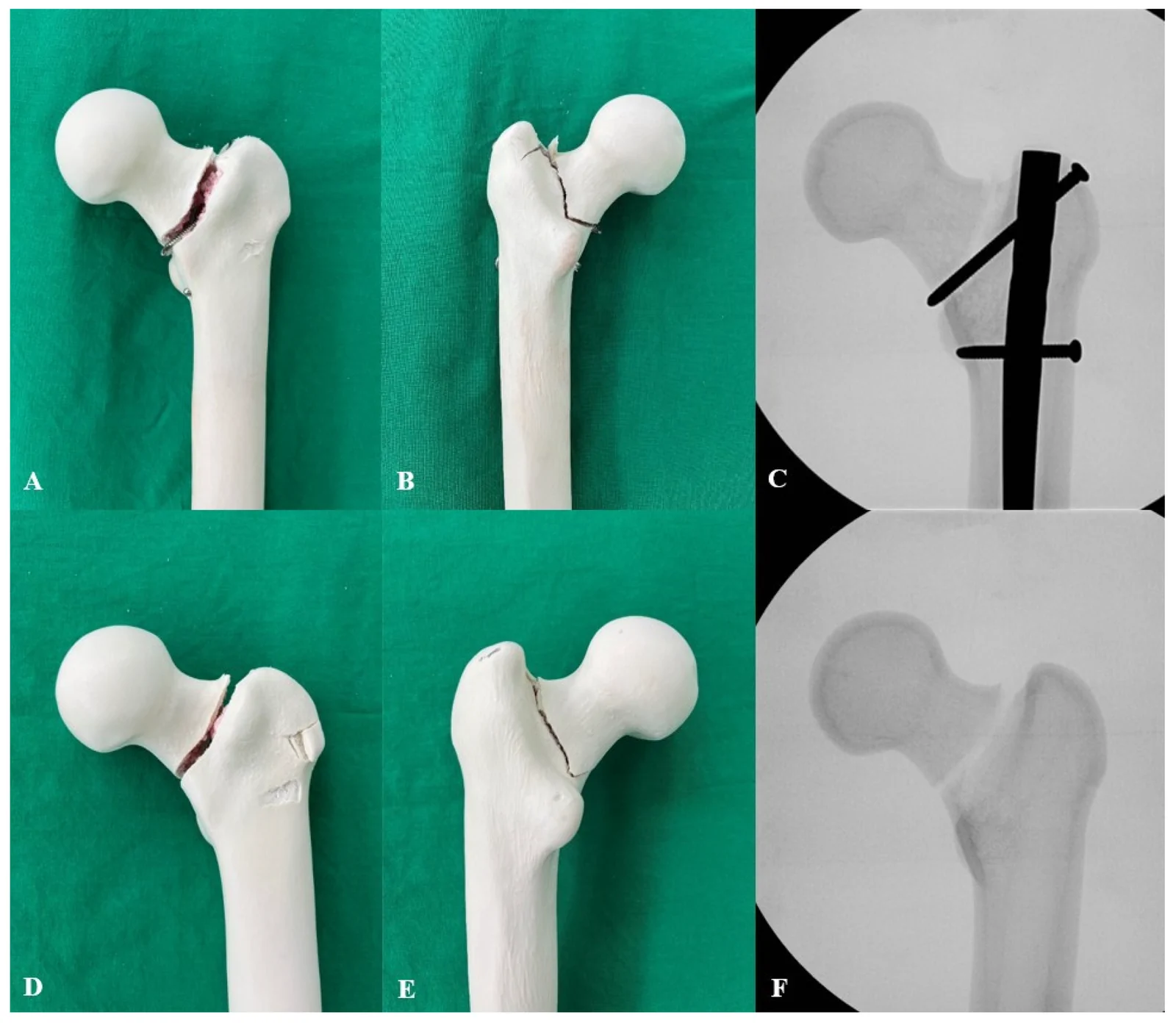
Anterior (**A**) and posterior (**B**) photographs of the specimen after the lateral impact test in group 3 with an anteroposterior C-arm image (**C**). Anterior (**D**) and posterior (**E**) photographs of the specimen after lateral impact test in the native synthetic femur with an anteroposterior C-arm image (**F**).

The fracture patterns of the specimens were investigated after the lateral impact test. In group 1, the main fracture line was observed in the basicervical area, and the fracture progressed to the greater trochanter but did not extend to the lateral cortex. The medial cortex of the basicervical area showed distraction with a visible gap, whereas the lateral cortex showed compression and collapse (Figure 6). C-arm examination revealed that the reconstruction screw was bent upward in the basicervical area (Figure 6). Group 2 had a fracture pattern similar to that of group 1, with a fracture in the basicervical area that did not propagate to the posterolateral cortex. Additionally, the fracture line invaded the anterolateral cortex in the direction of the GT tip (Figure 6). C-arm examination showed that the inferior reconstruction screw was slightly bent upward, and a fracture was observed in the GT area that was hollow due to the deep insertion of the IM nail (Figure 6). When comparing the reconstruction screws in groups 1 and 2, the single reconstruction screw in group 1 showed the greatest amount of bending, followed by the inferior reconstruction screw and the superior reconstruction screw in group 2 (Figure 7). In group 3, a fracture was observed in the basicervical area, and a distal fracture line was formed along the oblique screw (Figure 8). A linear fracture was observed in the basicervical area without comminution in the native synthetic femur (Figure 8).

In the axial cyclic incremental loading test, the mean number of cycles and total load to failure followed the order of group 2 (122,918.7 ± 1117.4 cycles, 164,650.9 ± 2737.7 kN), group 1 (81,531.4 ± 616.0 cycles, 79,680.0 ± 1078.0 kN), and group 3 (51,618.0 ± 569.1 cycles, 36,982.1 ± 697.2 kN). The overall differences among the three fixation constructs were statistically significant for both the number of cycles (*p* < 0.001, effect size η^2^ = 0.999, 95% CI: 0.998–1.000) and total load (*p* < 0.001, effect size η^2^ = 0.999, 95% CI: 0.998–0.999) (Figure 5). Post hoc analysis using Tukey’s HSD test revealed significant differences across all direct pairwise comparisons. Specifically, group 2 demonstrated a significantly greater number of cycles and total load than group 1 (both *p* < 0.001), and group 1 showed significantly greater values than group 3 (both *p* < 0.001).

Similarly, the initial axial stiffness was in the order of group 2 (553.6 ± 19.4 N/mm), group 1 (427.9 ± 8.1 N/mm), and group 3 (264.5 ± 9.7 N/mm), demonstrating a significant overall difference among the groups (*p* < 0.001, effect size η^2^ = 0.989, 95% CI: 0.973–0.993) (Figure 5). Subsequent direct comparisons via Tukey’s HSD test confirmed that the initial axial stiffness in group 2 was significantly higher than in group 1 (*p* < 0.001), which in turn was significantly higher than in group 3 (*p* < 0.001).

Furthermore, independent *t*-tests were conducted to compare these biomechanical properties against the native synthetic femur. Groups 1 and 2 had a significantly higher mean number of cycles, total load, and initial axial stiffness than the native synthetic femur (all *p* < 0.001). Conversely, group 3 exhibited a significantly lower mean number of cycles (*p* < 0.001), total load (*p* < 0.001), and initial axial stiffness (*p* < 0.01) compared to the native synthetic femur.

**Figure 9:**
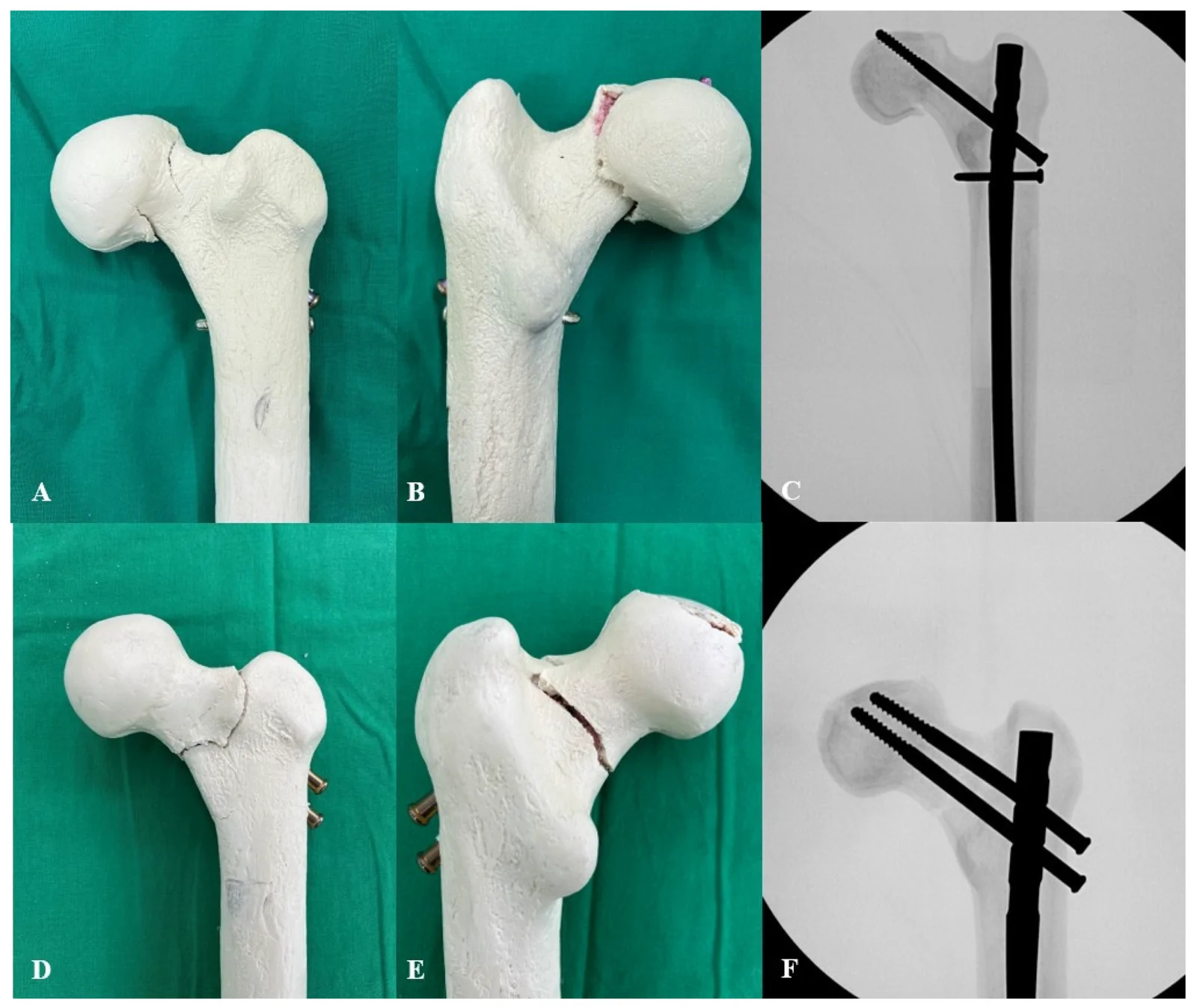
Anterior (**A**) and posterior (**B**) photographs and anteroposterior C-arm image (**C**) of the specimen after axial cyclic progressive loading test in group 1. Anterior (**D**) and posterior (**E**) photographs and anteroposterior C-arm image (**F**) of the specimen after axial cyclic progressive loading test in group 2.

**Figure 10:**
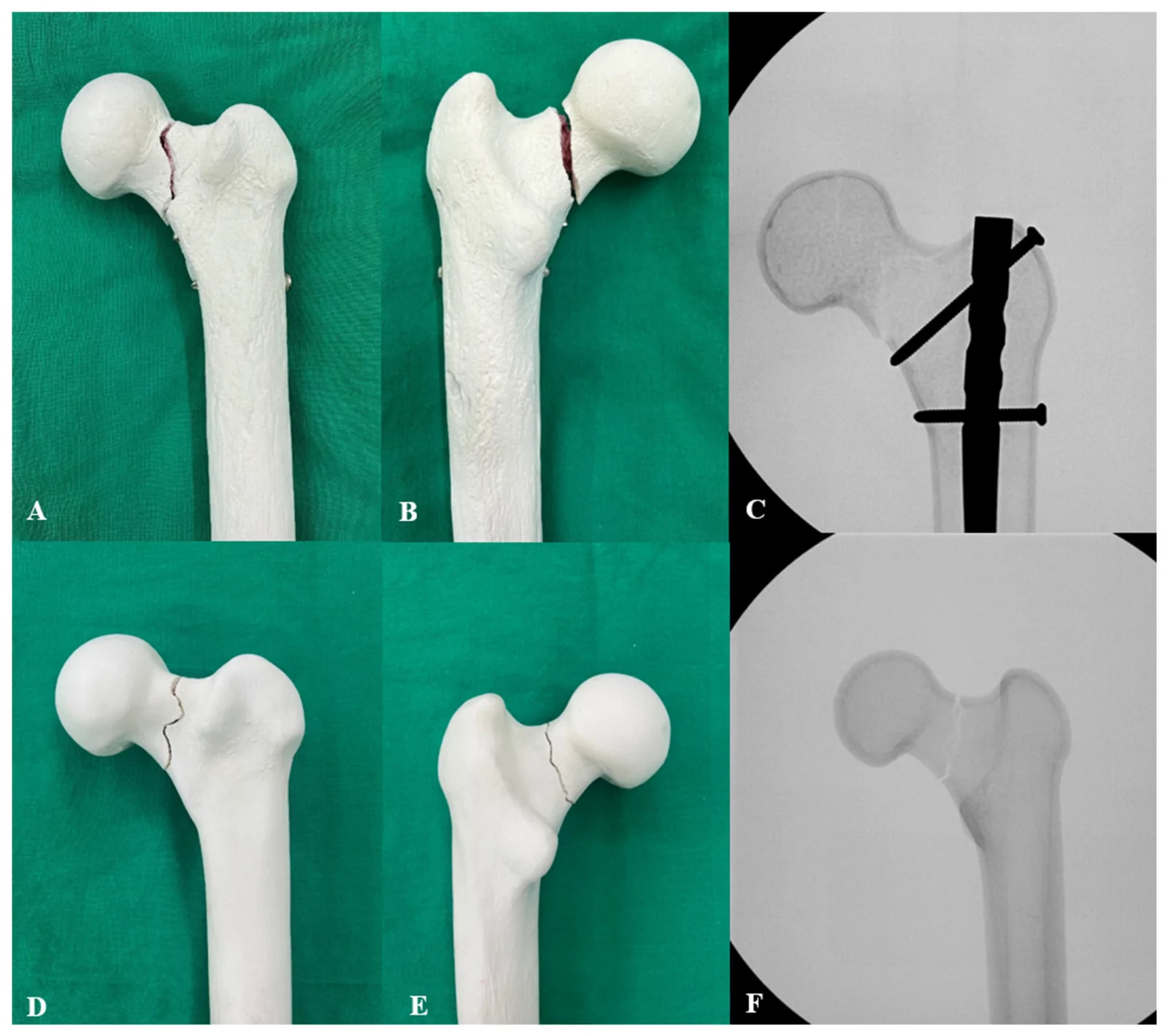
Anterior (**A**) and posterior (**B**) photographs and anteroposterior C-arm image (**C**) of the specimen after axial cyclic progressive loading test in group 3. Anterior (**D**) and posterior (**E**) photographs and anteroposterior C-arm image (**F**) of the specimen after axial cyclic progressive loading test in the native synthetic femur.

The fracture pattern in group 1 after the axial cyclic incremental loading test showed a vertical fracture line in the subcapital area and femoral head collapse with screw cut-out. No bending of a single reconstruction screw was observed on C-arm (Figure 9). In group 2, the femoral head collapsed, but screw cut-out did not occur, and a fracture line was observed in the basicervical area. The fracture line in the basicervical area was perpendicular to the reconstruction screw. On C-arm, the superior reconstruction screw was bent slightly downward in the basicervical area (Figure 9). No fracture lines were observed around the reconstruction screw or the inferior transverse interlocking screw insertion areas in the lateral cortex. In both group 3 and the native synthetic femur, a vertical fracture line was observed at the midpoint of the femoral neck (Figure 10). In group 3, the fracture line did not propagate along the oblique screw (Figure 10). In all cases, failure occurred in the femoral neck region, and no fracture was observed around the distal interlocking screws.

## 4. Discussion

To our knowledge, this is the first study to investigate biomechanical differences based on the number of reconstruction screws in a femoral IM nail using an osteoporotic femoral bone model. This study investigated the biomechanical properties of different cephalomedullary and proximal fixation methods. The results of this study demonstrated that both cephalomedullary fixations using two or one reconstruction screw showed a high degree of femoral neck protection, with two reconstruction screws being superior to one reconstruction screw. Proximal fixation using standard interlocking screws showed biomechanically inferior results compared with the native femur.

Low-energy femoral shaft fractures in elderly osteoporotic patients are associated with a relatively high risk of peri-implant fractures and subsequent revision surgery after IM nail fixation [12,42,43]. A previous study of 897 patients showed that in low-energy femoral shaft fractures, IM nail fixation with femoral neck protection resulted in no proximal peri-implant fractures, whereas the incidence of proximal peri-implant fractures without femoral neck protection was 5.4%, which was significantly different [12]. When treating femoral shaft fractures in elderly patients with IM nails, cephalomedullary fixation is recommended to protect the femoral neck, and cephalomedullary fixation with two reconstruction screws is commonly used.

Despite the need for cephalomedullary fixation, the use of two reconstruction screws is sometimes infeasible. Femoral neck width varies with race and sex, and studies have reported racial and sex difference of up to a 19%, with Asian women having the smallest width [21]. Other studies have also reported that the cross-sectional area of the femoral neck changes with age, decreasing by 7% per decade in women over 50 years [44,45]. Taken together, this suggests that elderly women, especially Asians, may have a narrow femoral neck, which may make cephalomedullary fixation with two reconstruction screws challenging. Further, the insertion of two reconstruction screws centered on the femoral head in the AP and lateral planes requires a learning curve and may increase the operative time and intraoperative radiation exposure [15,16]. These concerns have led to the use of only one reconstruction screw for cephalomedullary fixation, which is currently in wide clinical use [22,23]. Cephalomedullary fixation with a single reconstruction screw is believed to provide adequate protection to the femoral neck. However, limited biomechanical studies have been conducted to support this.

The results of this biomechanical study showed that cephalomedullary fixation with two reconstruction screws had an ultimate failure load and energy to failure in the lateral impact test of 3074.6 N and 40.4 J, respectively, while cephalomedullary fixation with one reconstruction screw had a failure load and energy to failure in the lateral impact test of 2681.4 N and 30.4 J, respectively. In a direct comparison between the two groups, the use of two reconstruction screws demonstrated significantly superior biomechanical outcomes than the use of one reconstruction screw across all parameters. This superior performance of two reconstruction screws over a single reconstruction screw is thought to be driven by higher construct stability, broader load transfer, and a more uniform stress distribution within the femoral neck. According to a recent cadaveric biomechanical study using an osteopenic hemi-pelvis, the unstable intertrochanteric fracture model using a single cephalomedullary lag screw exhibited significantly greater femoral head rotation and varus collapse than the model using dual cephalomedullary lag screws [46]. The study concluded that the larger surface area and integrated profile of the dual lag screws played a crucial role in enhancing construct stability and resisting torsional forces [46]. In our study as well, the use of two reconstruction screws increased the implant-bone contact area within the femoral neck, which likely influenced construct stability. Furthermore, the use of two reconstruction screws during the lateral impact test resulted in less screw deformation even at higher forces, and in the axial cyclic loading test, less screw cut-out was observed with two reconstruction screws (Figure 7 and Figure 9). This demonstrates that the forces applied with two reconstruction screws are distributed across two structures within the femoral neck, acting over a wider trabecular area and thereby mitigating focal stress concentrations.

Optimizing proximal fixation is particularly critical in the elderly osteoporotic population, where bone fragility frequently leads to catastrophic implant-related complications. Modern orthopedic trauma surgery has increasingly focused on broader, advanced strategies to manage these vulnerable patients. These range from biomechanical enhancements like cement augmentation for trochanteric fractures [47] and salvage arthroplasty utilizing megaprostheses for extensive bone defects [48], to complex soft tissue reconstructions for periprosthetic joint infections [49], and even the adoption of novel digital technologies like blockchain for secure patient data management [50]. Within this complex and evolving clinical landscape, our biomechanical findings underscore that maximizing initial mechanical stability—through optimal cephalomedullary screw configuration—remains fundamental to prevent devastating proximal peri-implant fractures and subsequent salvage procedures.

In this study, synthetic bone was used instead of human osteoporotic bone. Compared to previous cadaveric biomechanical studies, the synthetic femur bone model used in this study appears to be reliable. In a biomechanical study using elderly cadaveric femurs with a mean age of 69 years, the mean failure load at lateral impact was 2110 N [51]. Another study using cadaveric femurs with a mean age of 74 years reported an energy to failure of 5.5 J for lateral impact test [52]. A more recent cadaveric biomechanical study using female osteoporotic femurs with a mean age of 87.0 years reported a mean ultimate failure load of 1762 N and mean energy to failure of 10.2 J in a lateral impact test [53]. It should be noted that the results of the aforementioned studies all pertain to intact, non-implanted elderly cadaveric femurs [51,52,53]. For the native synthetic femur bone model used in the present study, the mean failure load in the lateral impact test was 1932.3 N and the mean energy to failure was 9.0 J, which is similar to previous reports using human cadaveric femurs. This suggests that both cephalomedullary fixation with two reconstruction screws and cephalomedullary fixation with one reconstruction screw can significantly increase the ultimate failure load and energy to failure during lateral impact and provide sufficient protection for the femoral neck. Although it offers relatively lower biomechanical stability than two reconstruction screws, a single reconstruction screw is considered a clinically feasible approach. It is thought to be particularly useful in patients with an anatomically narrow femoral neck (e.g., Asian elderly women), as well as for polytrauma patients or vitally unstable patients who require a short operative time. Conversely, if a patient’s vital signs are stable and the femoral neck width is deemed sufficiently wide to safely accommodate two reconstruction screws, the dual-screw configuration is strongly recommended.

IM nailing using standard interlocking screws without cephalomedullary fixation resulted in a 13.5% and 6.7% reduction in failure load and energy to failure in the lateral impact test compared with the native synthetic femur, and a 6.0% and 9.8% decrease in the number of cycles and total load in the axial incremental cyclic loading test, respectively. Fixation of the metal implant to the bone causes a sharp change in stiffness at the bone-implant interface, which leads to stress concentration and makes the interface region more prone to fracture [54,55]. The progression of osteoporosis in elderly patients results in decreased bone density and reduced bone stiffness, which maximizes stress concentration at the bone-implant interface [54,56,57]. According to a recently reported finite element study, a maximum equivalent stress of 76.7 MPa was observed in the femoral neck region when the IM nail was fixed with two proximal interlocking screws, whereas a maximum equivalent stress of 57.6 MPa was observed when two reconstruction screws were used in an osteoporotic femur, which showed a reduction of approximately 24.9% [56]. Based on the results of the present study and previous reports, proximal fixation using standard interlocking screws without protection of the femoral neck should not be performed in elderly patients with osteoporotic femurs because of the increased risk of peri-implant fracture.

In the lateral impact test, group 2 showed extension of the fracture line to the lateral cortex along the hollow GT tip. This is thought to be due to the GT tip area being unprotected by the implant, resulting in stress concentration at the bone-implant interface. If the IM nail is deeply positioned due to the difference in the neck-shaft angle of the implant and bone, an endcap of an appropriate length should be inserted at the proximal end of the IM nail flush with the GT tip to prevent fracture propagation. The insertion of the end cap is expected to result in a more even stress distribution around the GT tip; however, this has not been directly confirmed in the present study, and further research is needed to validate this hypothesis.

During the axial cyclic incremental loading test, a femoral neck fracture occurred in the subcapital area of group 1. This suggests that when a single reconstruction screw was inserted, the area adjacent to the nail body had a relatively high protective effect, but the femoral head area far from the nail body had a weaker protective effect, resulting in stress concentration at the head-neck junction. In group 2, fractures occurred in the basicervical area, with fractures formed in the direction perpendicular to the reconstruction screw inserted rather than in the direction of the applied force. C-arm examination revealed that the superior reconstruction screw was bent slightly downward with the apex located in the basicervical region. It is suggested that a relatively high amount of load was applied to the superior reconstruction screw, which caused it to bend, resulting in stress concentration at the apex of the bent screw. Moreover, the fracture propagated perpendicular to the screw owing to the micromotion of the screw.

No fracture line was observed around the screw insertion site after the axial cyclic loading test in groups 1 and 2. The distance between the proximal screw holes in group 1 was 5 mm, while that in group 2 was 8 mm. There is a case report of a stress fracture in the anterior cortex of the subtrochanteric region after IM nailing with a cephalomedullary blade and transverse interlocking screw for an incomplete atypical femoral shaft fracture [58]. This stress fracture is considered to have been caused by a difference in the radius of curvature (ROC) due to the use of a relatively straight nail to fix a femur with severe bowing, which may have concentrated stress in the proximal anterior cortex. Finite-element analysis of femoral stem revealed high stress on the proximal anteromedial cortex in a severely bowed femur model [59]. Taken together with other clinical studies that reported good results using a single reconstruction screw with a transverse interlocking screw [22,23], it is proposed that the ROC mismatch between the femur and IM nail may have contributed to stress fractures. The stress concentration effect is expected to be comparable in groups 1 and 2 owing to the similar distance between the proximal screw holes. Further research is needed on stress concentrations related to proximal screw configuration and ROC mismatch.

In both group 3 and the native synthetic femur, the fracture line occurred in the direction of the force applied to the middle of the femoral neck. This suggests that stress is concentrated in the narrowest region of the femoral neck when repeated physiological forces are applied [54].

This study has some limitations. First, the study used synthetic femurs rather than human cadaveric femurs, which may have influenced the results. However, the osteoporotic femur used in this study had similar biomechanical strengths to those reported in previous studies using human cadaveric femurs, which is believed to adequately reflect the properties of the elderly femur. In addition, the synthetic bone models had a uniform size, shape, and density, which may have led to more consistent results. Nevertheless, synthetic bones cannot fully replicate the natural biological variations found in cadaveric specimens, such as patient-specific trabecular architecture. Furthermore, real clinical scenarios involve dynamic biological processes, including bone healing, blood supply maintenance, and variable physiological loading over time. Therefore, accurately predicting how a single or two reconstruction screws will function in vivo, and determining the exact extent of any clinical complications, is difficult based on this biomechanical study alone. For instance, inherent surgical risks and complications associated with cephalomedullary fixation—such as osteonecrosis of the femoral head (ONFH), drill or screw protrusion into the hip joint, and subsequent hemarthrosis—have been reported in recent clinical reports [60,61,62]. Therefore, careful intraoperative execution and vigilant postoperative observation are required. Additional real-world clinical studies are warranted. Second, the synthetic femurs used in this study were osteoporotic models. The biomechanical properties of different proximal fixation methods in younger patients may differ from those in this study and warrant further investigation. Third, this study did not compare other types of implants, such as cephalomedullary screws and blades. This study aimed to investigate the biomechanical differences within a single type of implant. Future studies should investigate the biomechanical characteristics of other implant types. Fourth, because this study utilized intact femurs to simulate a healed femoral shaft fracture, it is not possible to extrapolate these findings to the acute postoperative period prior to callus formation, where an actual fracture site would represent a significant mechanical weak point. Fifth, this study did not consider the effects of soft tissues or muscles. The thickness of the soft tissue around the GT area affects the amount of force transmitted to the bone during fall [63,64,65]. In addition, adding a device that acts as an iliotibial band on the lateral side of the specimen during the axial cyclic loading test may provide more physiologically accurate results [39,66]. In future studies, adding devices that simulate soft tissues and muscles may provide more realistic results. Finally, there are differences between the protocols in this biomechanical test and actual falling and walking situations. Falls are common in situations such as stumbling. However, only a sideway-fall situation was simulated. During ambulation, the force applied to the hip joint changes by alternating between double- and single-leg stances. In this study, the hip joint was fixed in a single-leg stance position. Future biomechanical studies should include more diverse and detailed fall movements and gait postures.

## 5. Conclusions

Within the limitations of this intact synthetic bone model, cephalomedullary fixation with one or two reconstruction screws demonstrated superior relative mechanical performance compared to standard interlocking. The dual-screw configuration provided the highest stability, while a single reconstruction screw may serve as an acceptable biomechanical alternative when the placement of two screws is technically challenging. The use of proximal standard interlocking screws exhibited the lowest structural stability, indicating a higher biomechanical risk for stress risers and subsequent peri-implant fractures under similar loading conditions. Further studies with more physiologically accurate models and a wider variety of implants are required.

## Data Availability

The original contributions presented in this study are included in the article. Further inquiries can be directed to the corresponding author.
